# Recommendations for vaccination in multiple myeloma: a consensus of the European Myeloma Network

**DOI:** 10.1038/s41375-020-01016-0

**Published:** 2020-08-19

**Authors:** Heinz Ludwig, Mario Boccadoro, Philippe Moreau, Jesus San-Miguel, Michele Cavo, Charlotte Pawlyn, Sonja Zweegman, Thierry Facon, Christoph Driessen, Roman Hajek, Melitios A. Dimopoulos, Francesca Gay, Hervé Avet-Loiseau, Evangelos Terpos, Niklas Zojer, Mohamad Mohty, Maria-Victoria Mateos, Hermann Einsele, Michel Delforge, Jo Caers, Katja Weisel, Graham Jackson, Laurent Garderet, Monika Engelhardt, Niels van de Donk, Xavier Leleu, Hartmut Goldschmidt, Meral Beksac, Inger Nijhof, Niels Abildgaard, Sara Bringhen, Pieter Sonneveld

**Affiliations:** 1 Wilhelminen Cancer Research Institute, c/o 1st Department of Medicine, Center for Oncology, Hematology, and Palliative Care, Clinic Ottakring, Vienna, Austria; 2 Myeloma Unit, Division of Hematology, Azienda Ospedaliero-Universitaria Città della Salute e della Scienza, Torino, Italy; 3 Service hematologie et thérapie cellulaire, PRC. cic 1402 Inserm, CHU poitiers, Poitiers, France; 4 CIMA, IDISNA, CIBERONC, Clínica Universidad de Navarra, Pamplona, Spain; 5 Seràgnoli Institute of Hematology, Bologna University School of Medicine, Bologna, Italy; 6 The Institute of Cancer Research, London, UK; 7 Department of Hematology, Amsterdam UMC, VU University, Amsterdam, The Netherlands; 8 Hôpital Claude Huriez, Lille University Hospital, Lille, France; 9 Department of Medical Oncology and Hematology, Cantonal Hospital St Gallen, St Gallen, Switzerland; 10 Department of Hematooncology, University Hospital Ostrava and Faculty of Medicine, University of Ostrava, Ostrava, Czech Republic; 11 Hematology & Medical Oncology, Department of Clinical Therapeutics, National and Kapodistrian University of Athens, Athens, Greece; 12 Unité de Génomique du Myélome, University of Toulouse, Toulouse, France; 13 1st Department of Medicine, Center for Hematology, Oncology, and Palliatic Care, Clinic Ottakring, Vienna, Austria; 14 Department of Clinical Hematology and Cellular Therapy, Hospital Saint-Antoine, Sorbonne University, Paris, France; 15 IBSAL, Cancer Research Center, University Hospital of Salamanca, Salamanca, Spain; 16 Department of Internal Medicine II, University Hospital Würzburg, Würzburg, Germany; 17 Department of Hematology, UZ Leuven, Leuven, Belgium; 18 Department of Clinical Hematology, CHU of Liège, Liège, Belgium; 19 II. Medizinische Klinik und Poliklinik, Universitätsklinikum Hamburg—Eppendorf, Hamburg, Germany; 20 NCCC, Newcastle upon Tyne Hospitals Trust, Newcastle upon Tyne, UK; 21 INSERM, UMR_S 938, Centre de Recherche Saint-Antoine-Team Proliferation and Differentiation of Stem Cells, Assistance Publique-Hôpitaux de Paris, Hôpital Pitié Salpêtrière, Service d’Hématologie, Sorbonne Université, Paris, France; 22 Interdisciplinary Tumor Center, Faculty of Freiburg, University of Freiburg, Freiburg, Germany; 23 Poitiers University Hospital, Poitiers, France; 24 Internal Medicine V and National Center for Tumor Diseases (NCT), University Hospital Heidelberg, Heidelberg, Germany; 25 Department of Hematology, Ankara University, Ankara, Turkey; 26 Department of Hematology, Odense University Hospital, University of Southern Denmark, Odense, Denmark; 27 Erasmus MC Cancer Institute, Erasmus University of Rotterdam, Rotterdam, The Netherlands

**Keywords:** Health care, Disease prevention

## Abstract

Vaccination is one of the most successful medical interventions that has saved the life of millions of people. Vaccination is particularly important in patients with multiple myeloma, who have an increased risk of infections due to the disease-inherent immune suppression, and because of the immune suppressive effects of therapy. Hence, all appropriate measures should be exploited, to elicit an effective immune response to common pathogens like influenza, pneumococci, varicella zoster virus, and to those bacteria and viruses (haemophilus influenzae, meningococci, and hepatitis) that frequently may pose a significant risk to patients with multiple myeloma. Patients after autologous, and specifically after allogeneic transplantation have severely reduced antibody titers, and therefore require a broader spectrum of vaccinations. Response to vaccination in myeloma often is less vigorous than in the general population, mandating either measurement of the postvaccination antibody titers and/or repeating the vaccination. Here, we compile the existing data on vaccination in multiple myeloma and provide recommendations for clinical practice.

## Introduction

Infections remain the most common cause of morbidity and mortality in multiple myeloma besides the disease itself [1, 2]. The risk of infection is increased already at the stage of MGUS [3], and is even higher in patients with active disease when starting anti-myeloma therapy. One population-based study has estimated a tenfold higher risk for viral and a sevenfold higher risk for bacterial infections in multiple myeloma [4]. Severe humoral and cellular immune suppression, particularly during episodes of uncontrolled disease, account mainly for the increased susceptibility for infections. This predisposition is aggravated by the negative consequences of anti-myeloma therapy associated with severe immune suppression including impaired T-cell function and antibody production. Sensitizing the patient’s own immune system against frequent pathogens by vaccination during phases with no or little immunosuppression seems a logical approach in curbing the infection risk. Here, we review the risk of myeloma patients for infections possibly preventable by vaccinations, the available vaccines, their benefits and limitations, and provide recommendations for clinical practice.

## Methodology

Relevant literature published after 2000 was identified and reviewed using Medline, Cancerlit, and the Cochrane library. Recent studies presented at ASH, EHA, and EBMT were additionally taken into account. Following data extraction and assessment, a preliminary version was generated and revised by the authors. Comments and suggestions have been incorporated in the paper resulting in a final version, which has been approved by all authors.

Vaccination studies in myeloma are limited often by small numbers of enrolled patients and by laboratory outcome measures, which usually consist of evaluation of antibody titers only, and less frequently by proving clinical effectiveness (Table 1). Moreover, several recommendations are made in analogy to those made for the general population, or for similar diseases. International institutions, such as the center of disease control and prevention (CDC) [5], the World Health Organization (WHO) [6], or professional societies such as National Comprehensive Cancer Network (NCCN^®^) [7] and other published recommendations for vaccinations, either for the general population, for people aged 65 or older, for patients with impaired immune system [5], or for patients treated with autologous or allogeneic stem cell transplantation [7]. We needed to abstain from grading the recommendations for their strength of evidence, as suggested by the European Society of Microbiology and Infectious Disease [8], because of lack of data from randomized trials in patients with multiple myeloma. This is in accordance with a recent critical review of the Infectious Disease Society of America (IDSA) pointing to the suboptimal strength of scientific evidence for the majority of recommendations, which nevertheless are based on observational studies, clinical experience, and reports of expert committees [9].

**Table 1:** Phase II and phase III vaccination trials in patients with multiple myeloma.

Vaccine	# Of Patients/controls	Schedule	Outcome	Safety	Comments	Author
*Influenza*						
FV trivalent A/Singapore/6/86 (H1N1), A/Wuhan/359/95 (H3N2) and B/Beijing/184/93	MM: 52 patients 16 had chemotherapy <1 week and 7 had high-dose TX + ASCT <6 months prior to vaccination. 21 were on interferon α TIW	One dose	13% developed protective titers against one, 10% against 2, and 19% against all three strains	No AEs reported	Receiving chemotherapy <7 days prior to vaccination correlated with poor response	Robertson et al. [21]
FV trivalent A/California/7/09 (H1N1), A/Texas/50/12 (H3N2), and B/Massachusetts/60/ 08	15 pts with MM out of 36 with hematological malignancies and 70 with solid tumors	72% of all patients received a second vaccination	Protective titers to all three strains increased from 3 to 27%	A second vaccination in pts with no response recommended	Increase in protective titers varied between 3 and 10% dependent on strain	Sanada et al. [22]
FV trivalent A(H1N1)pdm09, iA(H3N2), B/Yamagata-lineage	48 pts with SMM or MM	50%	Protective titers to all three strains. 0 at baseline, 14% after first and 31% after second vaccination	A second vaccination in pts with no response recommended	Increase in protective titers varied between 18 and 44% dependent on strain	Hahn et al. [23]
*Herpes zoster*						
VZV gamma-irradiation inactivated	Scheduled for ASCT hematologic malignancies: 560/106 MM: 244/50 History of HVZ infection and/or antibodies to VZV	Four doses, first dose 5–60 days before ASCT, thereafter 30, 60, and 90 days after ASCT, or placebo	8 vs. 21% had HZV reactivation during 2.3–2.4 years FU	Proportion of individuals with injection site AEs 29 vs. 7%; General SAEs 33 vs. 33%	Patients had acyclovir or valaciclovir prophylaxis for 3 or 6 months after ASCT	Winston et al. [37]
Adjuvanted recombinant zoster glycoprotein E vaccine (Shingrix^®^)	Hematologic malignancies (excluding CLL on oral therapy) receiving or having just finished immunosuppressive TXT: 286/283 MM: 67/65	Two doses, 1–2 months apart, or placebo	VZV reactivation in 2 vs. 14 patients 80.4 vs. 0.8% had antibody response to glycoprotein E. Humoral and cellular immune response persisted at months 13	Proportion of individuals with pain at the injection site: 79.5 vs. 16.4%, fatigue: 58.3 vs. 27.2%; general SAEs 23.3 vs. 29.4%	Antiviral prophylaxis according to standards of participating centers Vaccine efficacy 87.2% 30.2% were excluded from per protocol group from immune response evaluation, short follow-up	Dagnew et al. [39]
*Pneumococci*						
PCV13	SMM: 20	One dose	60% responders after 1 month, 35% after 6 months, and 25% after 12 months	Not reported	40% had a positive opsonophagocytic test after 1 month, 30% after 6 months, and 25% after 12 months	Bahuad et al. [52]
PCV13	MM: 7 Controls: 18	One dose	IgG response in MM: 23.3% vs. 25.7% in controls	Not reported	Response 6 months after vaccination, MM: 14.3%, controls: 25.7%	Mustafa et al. [55]
PCV7 or PPV23 (1:1 randomization)	MGUS: 15 MM: 15, 11 of them on active therapy Waldenströms disease: 15, 1 on active therapy Controls: 20	Half received single dose of PCV7 or PPV23	IgG and particularly IgM antibodies were significantly lower in MM	Not reported	Opsophagocytic activity correlated with IgG antibody levels in MGUS and controls, but not in MM, and Waldenström’s disease	Karlsson et al. [57]
PPV23	MM: 60 pts vaccinated before ASCT Pts with preexisting high antibody levels were excluded	One dose	Response in 33% Response rate higher (73%) in pts with CR	No SAEs observed	Correlation with disease control and antibody response	Hinge et al. [54]
PPV23 (Pneumovax II)	MM: 52 patients 16 had chemotherapy <1 week and 7 had high-dose TX + ASCT <6 months prior to vaccination. 21 were on interferon α TIW	One dose	Response in 40% Fourfold increase in specific antibodies was recorded: 56%.	No SAEs observed	Poor antibody response was associated with higher sepsis risk	Robertson et al. [21]
PCV13 and PPV23	MM: 20 pts scheduled for induction TX Median time between both vaccinations: 21 days	One dose of PCV13 and PPV23 each	Protective IgG response in 60% antipneumococcal serum levels of IgG. IgG2, IgM, and IgA increased by 5.1-, 5.9-, 1.5-, and 3.8-fold	Not reported	Antibody levels decreased significantly at follow-up (28–502 days) in pts without ASCT but was even more pronounced in those receiving ASCT	Renaud et al. [72]
*Haemophilus influenzae*						
Haemophilus influenza type B	MM: 52 patients 16 had chemotherapy <1 week and 7 had high-dose TX + ASCT <6 months prior to vaccination. 21 were on interferon α TIW	One dose	Pre-vaccination protective ab titers: 45% Postvaccination: 75%, fourfold increase in ab titers in 41	No SAEs reported by patients	Receiving high-dose therapy and ASCT <6 months prior to vaccination correlated with poor response	Robertson et al. [21]

## Immune suppression in multiple myeloma

Efficient anti-infective defense requires a complex interplay between antigen recognition, antibody response, cellular defense, and humoral factors such as complement. Depressed antibody production against common pathogens has been documented in MGUS patients and is even more pronounced in patients with multiple myeloma, and particularly in those with active disease [10]. The immune dysfunction is further aggravated by varying degrees of lymphopenia, neutropenia, and reduced opsonization and functional impairment of phagocytosis and intracellular killing [10]. Myeloma therapy frequently contributes to cytopenia and immune suppression and may lead to significant disruption of mucosal barriers. Immune senescence may add to immune deficiency in the majority of myeloma patients diagnosed at higher age, as both antibody and cellular response have been shown to be impaired in elderly people [11, 12].

### Viruses

#### Influenza

Influenza virus usually is transmitted through droplets and contact with contaminated surfaces. A person may be infectious before and during symptoms. Influenza spreads around the world in yearly outbreaks, resulting in about three to five million cases of severe illness and about 290,000–650,000 deaths, particularly in young children and in elderly people [13], especially in those with comorbidities or severe immunosuppression. Hemagglutinin and neuraminidase expressed on the membrane of influenza virus are important targets of vaccines, because the former mediates binding of virus to target cells and entry into their genome, and the latter promotes binding to target cells and the release of progeny virus from infected cells [14].

Influenza viruses show a high mutational activity, which requires adaptation of the composition of vaccines in yearly intervals. The trivalent influenza vaccines comprise two serotypes of influenza A and one of influenza B. The quadrivalent vaccine comprises a second serotype of influenza B. Vaccination with influenza induces a humoral immune response against hemagglutinin and less pronounced against neuraminidase glycoproteins [15], particularly the latter protect against intracellular uptake when exposed to the same virus type [16]. Although exposure to live influenza virus creates a strong CD4 T-cell effector response, vaccines usually elicit poor cellular immunity [17]. Clinical studies show seroconversion after vaccination in about 70–80% of patients with malignancies [18, 19] and a Cochrane review on 2275 patients with malignancies revealed a significant, albeit limited reduction in mortality in cancer patients receiving different types of influenza vaccines [20]. In myeloma, a study published two decades ago reported poor response to influenza vaccine [21]. Only 19% of patients developed protective antibody titers to all three strains and 10% against two viral strains of the vaccine. In another study in patients with solid tumors and hematologic malignancies including 15 patients with multiple myeloma, protective titers were obtained in 27% of patients [22], with little further increase after a second boost. However, it is unclear whether a hemagglutinin titer of 1:40 is the right cutoff distinguishing between a clinically relevant protection or not. A more recent study by Hahn et al. [23] showed preexisting humoral immunity against one or more influenza serotypes in 9–19% of the patients. After a single vaccination, the frequency of patients with ‘sufficient’ titers to one strain rose by 20–40%. After a second vaccine boost, the number of patients with assumed protective titers nearly doubled. Two doses of influenza vaccine were automatically given 30 days apart in another study [24]. Rates of seroprotection against all three strains increased from baseline 4% to 49%, and 65% following one, and two doses, respectively [24]. Hence, as influenza antibody testing with the hemagglutination inhibition assay is not established as routine procedure, we recommend that patients without documented immune response should automatically be vaccinated twice within a 4-week interval [25]. No correlation between myeloma therapy or treatment intensity, and seroconversion has been observed in the study by Nordoy [18], while in the study by Branagan et al. [24] active disease requiring therapy, less than partial response, and conventional chemotherapy were associated with lower likelihood for a serological response.

Vaccination with trivalent and the newer quadrivalent influenza vaccines is recommended for all patients with MGUS, SMM, and multiple myeloma, their family members and their care givers [26]. The recommendation for patients is supported by WHO [27], CDC [28] and by NCCN^®^ [29], all of them propose influenza vaccinations in immunocompromised individuals (Table 2). Vaccinating health care providers in long-term care units is supported by the WHO [30], the CDC [31], and by data from a recent review [32], but studies documenting a significant benefit in acute care units are not available. The vaccination should be planned before the start of treatment [33] and before the beginning of a new influenza season, or in a period after the end of treatment with deep response to myeloma therapy.

**Table 2:** Recommendations for vaccination in patients with multiple myeloma.

Infections	Vaccine type	Recommendation	Doses	Supported by	Comments
Influenza	Trivalent or quadrivalent (strains selected according seasonal prevalence)	All patients, nonimmune family members, close contacts and HCWs	Two, if antibody response after 1st administration documented, 1, yearly	CDC NCCN	CDC recommends high-dose flu vaccine in people 65 years of age or older
Hepatitis A	Inactivated hepatitis A vaccine	Patients traveling to areas of high endemicity	2	NCCN	May test ≥1 month after last dose
Hepatitis B	Recombinant hepatitis B vaccine	Patients traveling to areas of high endemicity, behavioral/occupational exposure, hemodialysis	3	NCCN	May test ≥1 month after last dose, Revaccination in non-responder, consider booster dose if antibody level <10 IU/L, may retest every 5 years
Pneumococci	PCV13	All patients	1	CDC, IDSA, NCCN	Conjugated vaccine to a mutant diphtheria toxin induces T-cell response
	PPV23	>2 months, or 6–12 months after PCV13 according to other CDC	1–3 Repeat in 3 years	NCCN for pts <65 years at first dose	Polysaccharide vaccine, less immunogenic than PCV
Haemophilus influenzae	Haemophilus influenza type B conjugate	All patients	1	CDC, NCCN^a^	May test ≥1 month after last dose ^a^Also in patients traveling to endemic areas or in case of local outbreak
Meningococci	Meningococcal conjugate	Patients with asplenia, complement deficiency, recurrent episodes of bacterial infections	1	CDC, NCCN^a^	^a^Also in patients traveling to endemic areas or in case of local outbreak
Tetanus, diphtheria toxoids, and pertussis combined	Tetanus and diphtheria toxoids, and acellular pertussis	Patients who did not receive a primary vaccination for TDP, or a booster dose of tetanus and diphtheria toxoid vaccine. May be limited to tetanus only based on epidemiological prevalence	3	CDC, NCCN, WHO	May test for tetanus antibody titers at baseline and ≥1 month after last dose Booster dose of tetanus every 10 years
Herpes zoster	Recombinant VZV glycoprotein E vaccine (Shingrix^®^)	All patients with MM	2	EMN	Antibody response in 80.4%
	Live-attenuated VZV vaccine^a^ (Zostavax^®^)	All patients with MM	4	EMN	Estimated vaccine efficacy: 63%

*CDC* Center of Disease Control, *NCCN* National Comprehensive Cancer Network, *IDSA* Infectious Disease Society of America, *EMN* European Myeloma Network.

^a^Only in case recombinant VZV glycoprotein E vaccine is not available.

#### Varicella zoster

Patients treated with proteasome inhibitors [2], daratumumab [34], high-dose melphalan followed by autologous stem cell transplantation (ASCT) [2], and high doses of glucocorticosteroids [2] have a higher risk for varicella zoster virus (VZV) reactivation. Reactivation of latent virus residing in sensory ganglions leads to active virus production, spread alongside the axons down to the area of skin innervated by that ganglion, inflammation, blisters, and pain [35]. One large study on 9253 myeloma patients reported a hazard ratio of 14.8 for VZV reactivation compared to the general population [4]. Vaccines consisting of live-attenuated herpes virus [36], although found to be safe and active in myeloma patients, were generally not recommended in patients with multiple myeloma with frequently impaired immune system. Inactivation of VZV precludes any potential risk of reinfection. A recent randomized study with a gamma-irradiated and thereby inactivated VZV vaccine was found active in a large group, including around 45% with myeloma of autologous stem cell transplant recipients, dosed once 5–60 days before and three times (30, 60, and 90 days) after ASCT. The vaccination reduced VZV reactivations (8 vs. 21%) significantly during a 2.3–2.4 year follow-up period) and postherpetic complications. The vaccine was safe with no difference in AEs other than in local injection site reactions [37]. A new adjuvanted recombinant VZV glycoprotein E vaccine reduced the risk for VZV reactivation by 97% in elderly individuals of the general population and was well tolerated [38]. A trial with two doses of this vaccine in patients with hematological malignances, including 132 patients with multiple myeloma during or after immunosuppressive therapy, showed a humoral immune response in 80.2% of vaccinated patients with practicably no conversion (0.8%) in those receiving placebo [39]. Only two patients of the active study drug group developed VZV reactivation, as compared to 14 of the placebo arm during a 13 months follow-up period.

Based on these results, a proactive position favoring the use of the recombinant VZV glycoprotein E vaccine over the live-attenuated VZV vaccine seems justified practically in all patients with multiple myeloma, as many of them will receive ASCT and almost all treatment with immunosuppressive drugs. Patients should receive two doses 2–6 months apart. This strategy should be complemented by conventional prophylaxis with acyclovir or valaciclovir for further risk reduction. This is of particular importance in patients receiving proteasome inhibitors, or anti-CD38 antibodies.

#### Hepatitis A virus

Vaccination with an inactivated hepatitis A vaccine is recommended in nonimmune patients traveling to endemic regions (Southeast Asia, Mediterranean countries, Africa, Middle and South America). Patients should receive two doses given at least 6 months apart to achieve long lasting protection [40], which can be attained in up to 95% of the general population. Prophylaxis with intravenous immunoglobulins is an alternative option for those exposed for only a limited period. Protective anti-hepatitis A antibody titers have been detected in all five immunoglobulin products tested [41, 42].

#### Hepatitis B virus

Patients planned for therapy with proteasome inhibitors, immunomodulatory drugs, high-dose dexamethasone, monoclonal antibodies, and/or stem cell transplantation should be screened for hepatitis B by testing for HBs-Ag and anti-HBc antibodies [43]. In case of negative HBs-Ag and positive anti-HBc results, patients should be tested for hepatitis B DNA. In patients without evidence of hepatitis B infection, no further action is needed unless the patient lives in, or travels to, areas endemic for hepatitis B or patients who have sexual partners with chronic hepatitis B infection. In those individuals, vaccination with hepatitis B is recommended. In case of HBs-Ag positivity and/or positive hepatitis B DNA, antiviral therapy should be administered [41] concomitantly to anti-myeloma treatments with significant T-cell immunosuppressive activity. The third generation antiviral nucleoside analogues entecavir or tenofovir, are highly active, with almost no risk for developing resistance and are well tolerated, and thus recommended for those patients [44, 45]. Antiviral prophylaxis should be continued for several months and preferably until completion of anti-myeloma therapy, but exact figures from randomized trials are not available. Up to now, the newer and possibly more potent antiviral drugs such as entecavir, adefovir or tenofovir have not systematically been studied in multiple myeloma, but it can be anticipated that those new drugs will be associated with a lower risk for resistance development.

#### Hepatitis C virus

The same diagnostic procedures recommended for patients subjected to highly immunosuppressive therapy should be applied for hepatitis C screening. Patients with detectable disease documented by hepatitis C virus RNA should receive therapy with direct-acting antivirals, if possible before start of myeloma therapy. In spite of intensive research, there is no vaccine available today [46].

#### Measles, mumps, and rubella

Vaccination against measles, mumps, and rubella (MMR) is routinely provided by most health care systems of developed countries and there are no reports indicating that patients with multiple myeloma are at greater risk than the general population to develop clinically relevant disease induced by the aforementioned viruses. MMR vaccination usually leads to live long protection, but antibody levels and avidity may wane by up to 25% after 20 years of follow-up [47]. Nevertheless, after allogeneic transplantation, the probability of becoming seronegative was 60% for measles, 73% for mumps, and 52% for rubella in one study [48], which mandates either revaccination of those patients after a safety time period (>24 months) after transplantation or antibody testing against these viruses. Current available vaccines are live-attenuated and should not be used during the first 2 years following ASCT [49].

### Bacteria

#### Pneumococci

Streptococcus pneumonia species are common members of the bacterial flora colonizing the mouth and throat in 5–10% of healthy individuals and are a leading cause of pneumonia, otitis media, blood stream infection including sepsis and bacterial meningitis. The risk for developing a pneumococcal infection is increased in individuals with reduced IgG synthesis, impaired phagocytosis and defective bacterial clearance and higher age [50]. Over 90 serotypes have been identified [51], and two types of vaccines are commonly used to protect against the most important serotypes. The conjugate PCV13 (13-valent pneumococcal conjugate vaccine) vaccine contains purified capsular polysaccharide from 13 serologic strains conjugated to a mutant of diphtheria toxoid (CRM197). The coupling of the T-cell independent pneumococcal polysaccharide antigens to a carrier protein transforms them into T-cell dependent antigens [52] that are more immunogenic than those of the polysaccharide vaccine PV23. This latter vaccine induces antibody production in a T-cell independent manner by acting directly on B cells resulting usually in less efficient antibody production compared to T-cell dependent PCV vaccines.

Pneumococci vaccines have been evaluated in the general population, and in MGUS, SMM, and MM patients. A large randomized study employing a PCV13 vaccine showed 45% fewer episodes of vaccine-type community-associated pneumonia and invasive infections in adults aged ≥65 years [53]. Subsequent to this finding, conjugate vaccines have become the vaccine of choice in elderly individuals. In myeloma patients, substantial antibody responses were observed in about 30–60% of cases to either PCV13 or PPSV23 vaccines. In one study, a response to PV23 vaccination before ASCT was noted in 33% of patients, with a response rate of 73% in those who achieved a CR [54]. Similarly, in another trial, higher antibody responses were reported in patients with well-controlled disease, but antibody titers decreased within few months in most patients [52], an observation reported in another series as well [55]. In MGUS, a high response rate to a PCV13 vaccine was found [56], and in one of these studies, a reasonable correlation between IgG antibody response to four serotypes and opsonophagocytic capacity, but this correlation was not seen in patients with MM [57], where IgG antibodies without opsonophagocytic capacity were observed as well [10]. A study in elderly individuals of the general population aged 70 years or older showed that antibody response to several serotypes can be boosted by using higher doses of PCV7, but this benefit was associated with more adverse events [58], and data supporting this strategy in patients with multiple myeloma are not available as yet. As both PCV13 and PPSV23 vaccine were found to protect against pneumococcal disease in the general population [59, 60], patients should be vaccinated with PCV13 (in case of no previous PCV13 vaccination) followed by PCV23 after 2 months or an even longer interval. PPSV23 vaccination should be repeated in 5-year intervals but antibody response to a boost with PPSV23 may be lower than after primary vaccination [51].

#### Haemophilus influenzae

Haemophilus influenzae is a common gram-negative human-restricted bacterial pathogen that frequently colonizes the nasopharynx. It can cause local infections, such as otitis media and sinusitis and after breaching the epithelial barriers, it can result in invasive disease, including pneumonia, meningitis, and sepsis [61]. More than 50% of patients with MM lack protective anti-haemophilus influenzae (Hib) antibodies and serum bactericidal activity against Hib is absent in 70% of patients [62]. Vaccination with haemophilus influenzae is recommended in all patients with asplenia and should be considered in patients with MM, although data on the clinical efficacy are limited. One study showed antibody response in 71% of patients vaccinated after ASCT [63].

#### Meningococci

Low immunoglobulin levels, complement deficiencies, asplenia, and the diagnosis of multiple myeloma are among other well established risk factors for meningococcal disease [64]. One study reported a hazard ratio of 16.6 for patients with MM for developing meningococcal disease compared to controls [2].

Tetravalent capsular polysaccharide conjugate vaccines with improved immunogenicity targeting serotypes A,C,Y,W-135, and protein-based, capsular polysaccharide-free recombinant vaccines targeting serogroup B [64] are available for clinical use. The latter are now widely used for population vaccination programs as serogroup B accounts for the majority of cases [65].

An expert group [8] recommended to use the conjugated tetravalent conjugated vaccine and to consider vaccination against serogroup B as well in asplenic patients with hematological diseases. This seems reasonable in patients with MM in general, and particularly in those with splenectomy [66], complement deficiency and possibly also in the post-hematopoietic stem cell transplant setting.

#### Diphteria, pertussis, tetanus, and polio

Data on the relevance of vaccination with these pathogens are scarce in conventionally treated patients with MM. In lymphoma and AML patients, significantly more patients lacked protective antibody titers against tetanus and diphtheria after intensive therapy [67]. In patients receiving an allotransplantation a significant loss of antibody protection against tetanus and polio was reported [68, 69]. Pertussis may cause infections in adults, but data on reappearance of pertussis in myeloma patients are limited; noteworthy, protective antibody titers often are decreased in patients after allotransplantation [70]. Overall, there is little information on the immunity against these pathogens in myeloma patients, but vaccination with these antigens is recommended in patients after allogeneic transplantation.

#### Autologous and allogeneic transplantation

Antibodies for various pathogens including pneumococci, haemophilus, and measles are significantly reduced after autologous [71, 72] and more so after allogeneic stem cell transplantation [73]. Transplanted patients have a higher risk for virus reactivation and develop a more severe course of viral infections [74]. These and other findings prompted the IDSA to consider recipients of allotransplantation as never vaccinated [75], highlighting the need for a full vaccination program. Several recommendations have been published by international and national societies for patients after stem cell transplantation [48, 49]. The NCCN^®^ Guidelines for patients after autologous and allogeneic stem cell transplantation are shown in Table 3. Patients should be vaccinated with vaccines administered during early childhood, and pneumococci, haemophilus influenzae and meningococci, influenza and recombinant VZV vaccine [29]. Due to the poor immune response after allogeneic transplantation, vaccination should be repeated in short intervals (4-week intervals) for most pathogens. The CDC recommends vaccination with MMR in patients 24 months after allogeneic transplantation and without signs of graft versus host disease [5]. There is some inconsistency regarding the appropriate timing of the vaccinations. As patients are prone to increased infection risk shortly after transplantation, some experts recommend to administer seasonal influenza vaccine already a few months after ASCT. A second vaccination should be considered to increase antibody response [76]. Timing vaccination relatively early after transplantation is supported by data showing comparable response rates to PCV vaccination in patients who were vaccinated 3 or 9 months after the transplant [77]. Hence, there are relevant arguments for starting some vaccinations, like influenza and PCV13, already about 3 months after autologous transplantation, but it should be kept in mind that official organizations take a more conservative approach favoring a longer interval between autologous transplantation and vaccination as shown in Table 3 [29].

**Table 3:** Recommended vaccination schedule after autologous or allogeneic HCT (with permission of the NCCN^®^).

Inactivated vaccines^a^	Recommended timing after HCT	Number of doses
DTaP (diphtheria/tetanus/acellular pertussis)	6–12 months	3
Pneumococcal vaccination		
• Conjugated 13-valent vaccine	6–12 months	3
• Upon completion of PCV13 series, then PPSV23	≥12 months	1
Hepatitis A^b^ (Hep A)	6–12 months	2
Hepatitis B^b^ (Hep B)	6–12 months	3
Meningococcal conjugate vaccine^c^	6–12 months	1–2
Influenza (injectable)^d^	4–6 months	1^d^, annually
Inactivated polio vaccine	6–12 months	3
Recombinant zoster vaccine	50–70 days after autologous HCT May be considered after allogeneic HCT^e^	2
Human papillomavirus (HPV) vaccine	>6–12 months For patients up to age 26, consider up to age 45	3
Live vaccines	Recommended timing after HCT	Number of doses
Measles/mumps/rubella (MMR)^f^	≥24 months (if no GVHD or ongoing immunosuppression and patient is seronegative for measles, mumps, and/or rubella)	1–2
Varicella vaccine^f^	≥24 months (if no GVHD or ongoing immunosuppression and patient is seronegative for varicella)	1
Zoster vaccine^f,g^ (category 3)	May be considered at ≥24 months (if no GVHD or ongoing immunosuppression)	1

Reproduced with permission from the NCCN Guidelines^®^ for prevention and treatment of cancer-related infections V.1.2020. ^©^2019 National Comprehensive Cancer Network, Inc. All rights reserved. The NCCN Guidelines and illustrations herein may not be reproduced in any form for any purpose without the express written.

^a^Inactivated vaccines may be given as a combined vaccine. Vaccination may be postponed for patients receiving >20 mg of prednisone.

^b^Strongly consider if clinically indicated. May consider Hep A and B combined vaccine if immunization for both is needed.

^c^Meningococcal B vaccine should be considered for high-risk patients such as patients with asplenia or complement deficiency or patients receiving eculizumab.

^d^As antibody response may be suboptimal, EMN recommends a second administration, or confirmation of antibody response by adequate testing.

^e^Efficacy in allogeneic HCT, in the presence of GVHD, or ongoing immunosuppression has not been established (Bastidas A, et al. JAMA. 2019;322:123–33).

^f^MMR and varicella/zoster vaccines may be given together or 4 weeks apart.

^g^Because of insufficient data on safety and efficacy of live zoster vaccine among HCT recipients, physicians should assess the immune status of each recipient on a case-by-case basis and determine the risk for infection before using the vaccine. Randomized data exist for use of the recombinant zoster vaccine in patients receiving autologous HCTs but not for the live zoster vaccine.

#### Monoclonal antibodies, T-cell engagers (BiTEs), CAR-T cells

A small series of patients with RRMM treated with daratumumab showed a similar vaccination response to PCV13 and PPV23, Haemophilus influenza, and seasonal influenza compared to patients receiving non-daratumumab containing regimes [78]. Similar responses likely are to be expected for other CD38 monoclonal antibodies. No information is available for the other above-mentioned treatments. Patients subjected to treatment with BiTEs or CAR-T cells usually are heavily pretreated and often present with severely compromised bone marrow reserve and long lasting impairment of anti-infective immune response. Ideally, patients should be vaccinated before start of rescue therapy with the entire spectrum of vaccines listed in Table 2, with influenza, VZV, and vaccination against encapsulated bacteria (pneumococci, haemophilus influenzae, and meningococci) being most important.

#### Disease status and vaccination

Common reasoning suggests that patients should be vaccinated before transformation into active myeloma at MGUS or SMM stage, or during remission when there is no or only minor immune suppression by active disease, but scientific support for this notion is available in CLL patients only [79]. Limited evidence suggests that patients on lenalidomide maintenance show an enhanced antibody response from the immune stimulatory effects induced by IMiDs [80], but a recent study in patients on lenalidomide maintenance was unable to confirm this [63]. In patients on daratumumab therapy, a similar vaccination response was reported compared to patients receiving non-daratumumab containing therapy [78]. Patients with scheduled chemotherapy should be vaccinated at least 2 weeks before initiation of chemotherapy [5], upon achievement of best response, 3–6 months after completion of chemotherapy or autologous transplantation, and 6–24 months after allogeneic transplantation. Vaccination with inactivated or live vaccines should not be given either before or about 3 months after treatment with intravenous immunoglobulin therapy because of concerns about effectiveness of vaccines [5].

#### Family members, close patient contacts, and health care workers

Persons in close contact with the patient should receive all age- and exposure appropriate vaccines and should specifically be vaccinated against influenza, and those aged 65 years or older against pneumococci (Table 4). Patient contacts should be aware that there is a small risk of transferring live vaccines with the exception of MMR [5] to the patient. Studies from several countries show significant immunity gaps against many vaccine-preventable diseases in health care providers highlighting the need for uniform recommendation. Immunity against the spectrum of pathogens vaccinated against during childhood should be ascertained. This is particularly relevant for mumps, rubella, and measles [81, 82]. In case of inadequate antibody response, revaccination is recommended. In addition, health care workers should be vaccinated against hepatitis B, and regularly against influenzae, and depending on specific situations against other pathogens listed in Table 4.

**Table 4:** Recommendations for vaccination of family members and for health care personnel.

Vaccine	Dose	Family member	Health care personnel	Supported by
Influenzae	One dose, assess response and repeat if insufficient (or use two doses without response assessment)	+++	+++	CDC
Hepatitis A	One dose, administer booster dose 6–36 months later	Only if traveling to endemic areas	+	
Hepatitis B	One dose, assess response within 1–6 months, or administer three doses without assessment	If patient has active disease^a^ or plans traveling to endemic areas or has a sexual partner with chronic hepatitis B infection	+++	CDC
Pneumococci	PCV13 followed after ≥2 months by PV23	If age 65 years or older	If age 50 years or older^b^	EMN
Meningococci	One dose with a booster every 5 years	−	Personnel handling N. meningitis specimen	CDC
Varicella	Two doses 4 weeks apart	−	+++ If no preexisting immunity	CDC
Varicella zoster virus	Two doses of recombinant glycoprotein E	If age 65 years or older	If age 50 years or older^b^	EMN
Diphtheria, tetanus, pertussis	One dose if not received in adulthood Get booster ever 10 years (even if preexisting immunity)	+ If no preexisting immunity	+++ If no preexisting immunity	CDC
Mumps, measles, rubella	Two doses if no evidence of immunity	+ If no preexisting immunity	+++ If no preexisting immunity	CDC

^a^HBV-DNA, HBs-AG, or HBe-Ag positive.

^b^May be considered, *CDC* Center of Disease Control, *NCCN* National Comprehensive Cancer Network, *EMN* European Myeloma Network, − not recommended, + recommended, +++ highly recommended.

#### Contraindications, precautions, and side effects

Severely immunosuppressed patients should not receive live vaccines. Myeloma patients, if not in sustained well-controlled remission, are considered immunosuppressed, and thus are not candidates for life vaccines. Vaccination should also be withheld in an individual who had a severe allergic reaction after a previous dose or vaccine component. Vaccination should be deferred in patients with uncontrolled disease, ongoing infections, or other acute illnesses. Presently, there are no contraindications for vaccinating patients with a previous episode of infection with the same class of pathogens the vaccine should provide protection for. For example, vaccination with PCV13 is recommended in a patient even if he had a previous episode of pneumococcal pneumonia, because the vaccine contains serotypes the patient might not have been exposed to.

Modern vaccines usually are very well tolerated. Local reactions, such as areas of redness, swelling, pain, and infrequently induration, may occur at the injection site. Such reactions may increase in severity with each subsequent injection. Very rarely, general reactions such as fever, chills, feeling tired, headache, muscle and joint aches are encountered. Neurological side effects, including Guillain–Barre syndrome (after influenza vaccination), anaphylaxis, bronchospasm, laryngeal edema, generalized collapse, and prolonged unresponsiveness have previously been reported as very rare complications with an incidence of about 1/1,000,000 [83, 84].

## Limitations

Many of the available data have been published before the introduction of novel myeloma drugs and treatment strategies, which nowadays result in higher response rates and deeper responses, reducing or even obviating myeloma induced immune suppression. Several studies included small patient numbers, making it difficult to evaluate the validity of their findings. Almost all studies reporting response to vaccination provide data on antibody response only; there is no validation whether a rise in antibody titer to ≥1:40 correlates with the assumed clinical protection, nor are data available on the impact of opsonophagocytic activity on clinical efficacy. Cellular immunity is usually not assessed. Randomized trials providing the scientific evidence for vaccination recommendations in multiple myeloma are available for a few indications only; most of the recommendations rely on clinical observations, consensus of experts, published by international or national societies, observational studies, and on analogies drawn from the general population. In spite of these limitations, thorough review of the existing data and balancing the weight of evidence within a group of experts of the disease likely provides relevant information for clinical care. This was our main objective, which we hope to have accomplished.

## Conclusion

Infections are the second major cause of mortality in multiple myeloma, mandating optimization of measures for prevention of infections. Vaccination is one of the greatest achievements of medical research, which has saved the life of millions of people, and an effective preventive strategy in patients with multiple myeloma. Ongoing research has led to the development of genetically engineered VZV vaccines obviating the need of using attenuated live vaccines, and further improvements in efficacy and safety of other vaccines can be expected for the near future. Still, in real world clinical practice, the entire potential of a comprehensive vaccination policy is underused. This work aims to provide the necessary medical background and recommendations for an optimal vaccination strategy in myeloma patients likely to benefit from this important preventive measure.
